# Seasonal epidemiology of gastrointestinal nematodes of cattle in the northern continental climate zone of western Canada as revealed by internal transcribed spacer-2 ribosomal DNA nemabiome barcoding

**DOI:** 10.1186/s13071-021-05101-w

**Published:** 2021-12-11

**Authors:** Tong Wang, Elizabeth M. Redman, Arianna Morosetti, Rebecca Chen, Sarah Kulle, Natasha Morden, Christopher McFarland, Hannah Rose Vineer, Douglas D. Colwell, Eric R. Morgan, John S. Gilleard

**Affiliations:** 1 Host-Parasite Interactions Program, Faculty of Veterinary Medicine, University of Calgary, Calgary, Canada; 2 School of Biological Sciences, Queen’s University Belfast, Belfast, UK; 3 Institute of Infection, Veterinary and Ecological Sciences, University of Liverpool, Liverpool, UK; 4 Agriculture and Agri-Food Canada, Lethbridge, Canada

**Keywords:** Gastrointestinal nematodes, Epidemiology, Grazing season, Internal transcribed spacer-2 ribosomal DNA metabarcoding, Microclimate

## Abstract

**Background:**

Gastrointestinal nematode (GIN) epidemiology is changing in many regions of the world due to factors such as global warming and emerging anthelmintic resistance. However, the dynamics of these changes in northern continental climate zones are poorly understood due to a lack of empirical data.

**Methods:**

We studied the accumulation on pasture of free-living infective third-stage larvae (L3) of different GIN species from fecal pats deposited by naturally infected grazing cattle. The field study was conducted on three organic farms in Alberta, western Canada. Grass samples adjacent to 24 fecal pats were collected from each of three different pastures on each farm. Internal transcribed spacer-2 nemabiome metabarcoding was used to determine the GIN species composition of the harvested larvae. The rotational grazing patterns of the cattle ensured that each pasture was contaminated only once by fecal pat deposition. This design allowed us to monitor the accumulation of L3 of specific GIN species on pastures under natural climatic conditions without the confounding effects of pasture recontamination or anthelmintic treatments.

**Results:**

In seven out of the nine pastures, grass L3 counts peaked approximately 9 weeks after fecal deposition and then gradually declined. However, a relatively large number of L3 remained in the fecal pats at the end of the grazing season. Nemabiome metabarcoding revealed that *Cooperia oncophora* and *Ostertagia ostertagi* were the two most abundant species on all of the pastures and that the dynamics of larval accumulation on grass were similar for both species. Daily precipitation and temperature across the whole sampling period were similar for most of the pastures, and multiple linear regression showed that accumulated rainfall 1 week prior to sample collection had a significant impact on the pasture L3 population, but accumulated rainfall 3 weeks prior to sample collection did not.

**Conclusions:**

The results suggest that the pasture L3 population was altered by short-term microclimatic conditions conducive for horizontal migration onto grass. Overall, the results show the importance of the fecal pat as a refuge and reservoir for L3 of cattle GIN on western Canadian pastures, and provide an evidence base for the risk assessment of rotational grazing management in the region.

**Graphical Abstract:**

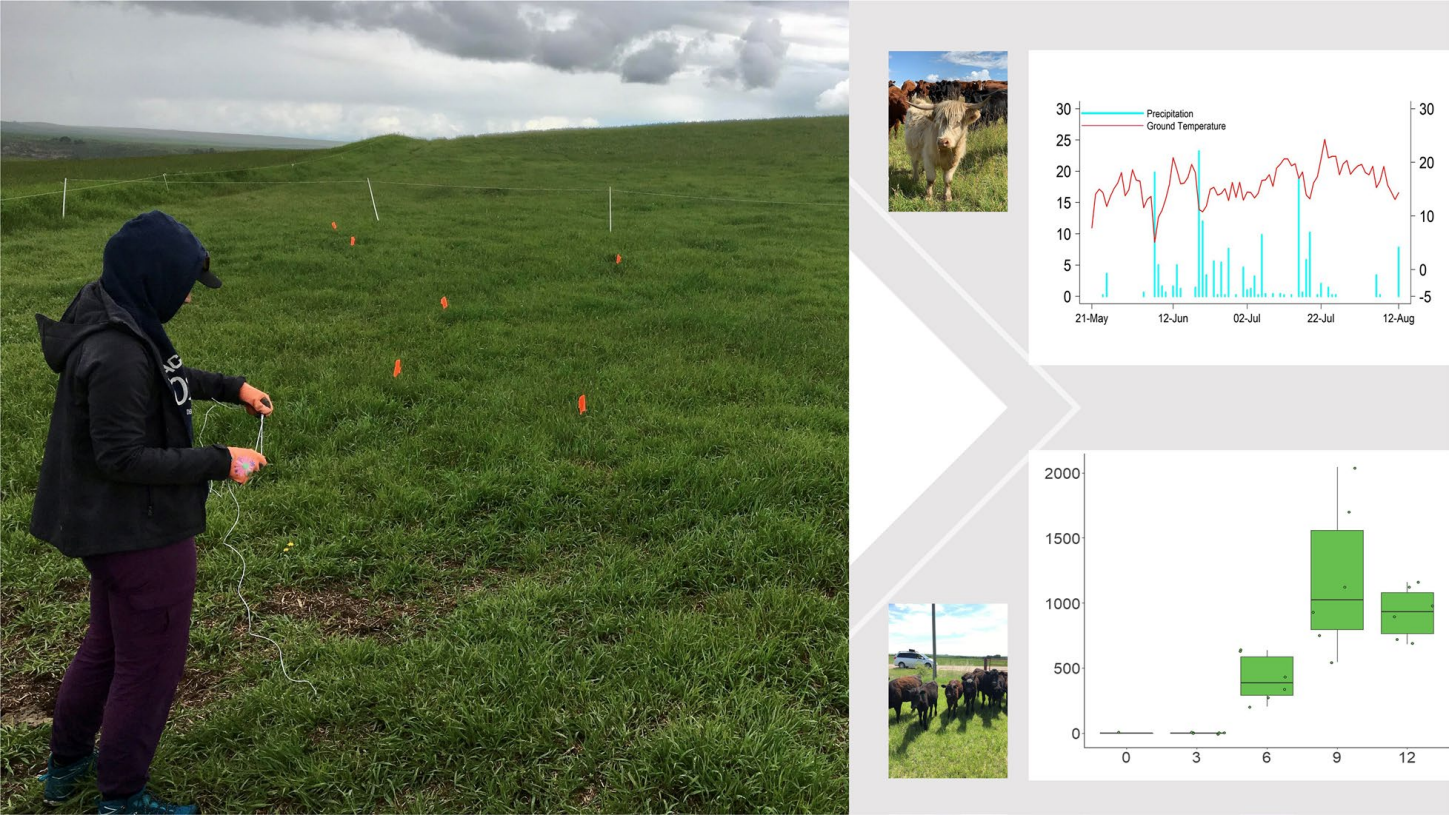

**Supplementary Information:**

The online version contains supplementary material available at 10.1186/s13071-021-05101-w.

## Background

Gastrointestinal nematodes (GIN) are amongst the most important pathogens of grazing ruminants worldwide, and have major negative impacts on animal health and production. In northern temperate regions, although clinical disease can occur in beef cattle that graze [1, 2], sub-clinical infection has a much greater impact and may go undetected by producers [3]. In temperate climate zones, the prevalence of cattle GIN is high, but the intensity of infection is generally lower than in sub-tropical and tropical regions [4]. However, GIN infection intensities appear to be increasing in temperate climate zones for a number of reasons, including climate change [5, 6] and the emergence of anthelmintic resistance [7, 8]. More northerly regions are particularly sensitive to these changes, and the epidemiology of cattle GINs in northern continental climate zones represents an important knowledge gap of relevance to the risk assessment and the implementation of evidence-based effective and sustainable GIN control practices.

A significant amount of work has been undertaken on the epidemiology of cattle GINs in temperate latitudes of the USA and Europe [9–12]. In temperate regions of the northern hemisphere, the infection of set-stocked cattle with overwintered GIN larvae begins after spring turn-out onto pasture, while inhibited larvae in older hosts also mature at this time [9, 13]. Eggs are produced during these early season infections which contaminate pasture and develop into infective third-stage larvae (L3), leading to a peak in adult worm abundance in mid-summer, prior to immunity-driven decline, while a proportion of the L3 linger on pasture and re-start the cycle the following year [14, 15]. However, much less is known about the epidemiology of cattle GINs in more northerly continental climates. These locations differ from more temperate regions in several important ways, including the occurrence of extremely cold and dry winters and drier summers with sporadic but sometimes heavy precipitation [16]. Recent work has shown that L3 of both *Ostertagia ostertagi* and *Cooperia oncophora* can survive over the winter in significant numbers on pastures in western Canada, despite the very low temperatures and low humidity at this time of the year, with the fecal pat being an important refuge [13]. Fecal egg counts are known to increase over the summer grazing season in beef cattle in western Canada, and there is some evidence that temperature and accumulated precipitation are significant predictors of *O. ostertagi* serum antibody concentrations [17, 18]. However, there is very limited information on the pasture dynamics of infective larvae over the grazing season in these northerly regions, and on whether there are differences between the major GIN species. These are important knowledge gaps since many of these regions, e.g. western Canada and northern USA, are major cattle grazing areas. Alberta alone possesses 41.6% of the Canadian beef cattle herd [19].

Co-infections of multiple GIN species are common in cattle worldwide, with species composition varying between regions [20, 21]. *Cooperia oncophora* and *O. ostertagi* are the two most prevalent GIN species in western Canada [8, 13]. *Ostertagia ostertagi* is the most pathogenic species in cattle, and although it can show anthelmintic resistance, reports of this are not common for North American beef cattle [22, 23]. In contrast, resistance to macrocyclic lactones has been documented in *Cooperia* species worldwide, including in North America [8]. Although *Cooperia* species are less pathogenic than *O. ostertagi*, they do cause production loss if poorly controlled [24], and are usually present in mixed infections with additive disease impacts [25, 26]. Given the differences in the pathogenicity, epidemiology and drug sensitivity that exist among GIN species, species-specific information is important both for routine diagnostics and epidemiological studies. Nemabiome metabarcoding is a recently developed approach used to determine the relative proportions of strongylid species in fecal or pasture larval samples, and is well suited to epidemiological or surveillance studies involving large numbers of samples [8, 13, 21].

In this paper, we present an investigation of the dynamics of pasture larval availability of the major GIN species in several beef cattle herds grazing over the summer in western Canada. The application of internal transcribed spacer (ITS)-2 nemabiome metabarcoding allowed the seasonal patterns of L3 of individual GIN species on pasture to be determined. The aim of this study was to determine the factors that influence the timing of L3 availability on herbage following the deposition of feces from grazing animals carrying natural mixed populations of GIN, and to provide support for evidence-based pasture management strategies.

## Methods

### Study sites

A field study was conducted on naturally infected pastures of three commercial organic beef cattle farms [located in Red Deer (farm 1), Waterton (farm 2) and Castor (farm 3), Alberta, Canada; 49°05′–52°16′N, 111°54′W–113°91′W]. None of the farms had used anthelmintics to treat GINs in the past 10 years. Each farm used an intensive rotational grazing pattern whereby the property was divided into small fields on which the herd sequentially grazed during the season (Table 1). Each herd was moved every 2 to 9 days to a new pasture that had not been grazed in the past 12 months, thus the cattle did not return to a pasture until the following grazing season. These features allowed us to monitor the seasonal ecology of free-living GINs under natural climatic conditions without the confounding effects of recontamination or anthelmintic treatment. We collected samples from three pastures on each farm, thus there were nine separate study pastures in total.

**Table 1:** Pasture and grazing information for the three study farms in Alberta, western Canada

Farm	Farm 1	Farm 2	Farm 3
Herd size	110 Yearlings	80 Yearlings	25 Yearlings and 110 cows
Pasture size (each field)	25–40 Acres	160 Acres	10 Acres
Rotation frequency	Every 2 days	Every 5 days	Every 7–9 days
Breed of cattle	Crossbred beef cattle	Crossbred beef cattle	Crossbred beef cattle

### Meteorological data

One solar-powered weather station (HOBO RX3000; Onset, MA) was set up on each farm, with probes at 1 cm above ground level (which is where GIN larvae reside on herbage). Temperature and precipitation data were recorded every 15 min and transferred hourly to HOBOlink Web-based software (https://www.hobolink.com/) with which the latest data could be downloaded. We chose to measure temperature closer to the ground than is typical for weather stations as this better represents the microclimate experienced by GINs in fecal pats and on pasture [27].

### Experimental design and environmental sampling

**Fig. 1:**
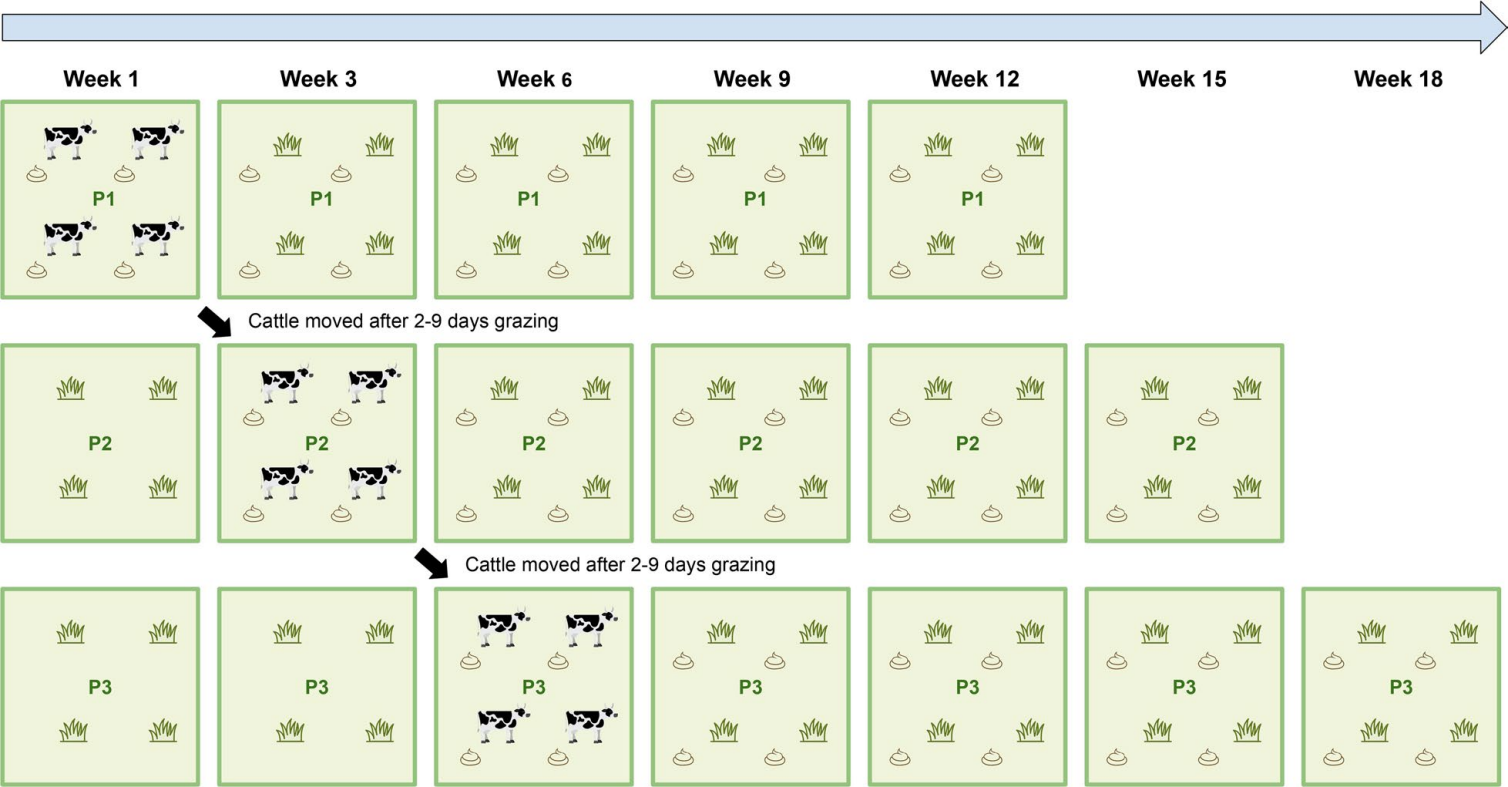
Experimental design for all three farms. Fresh feces containing gastrointestinal nematode eggs were deposited on each pasture for 2–9 days (depending on farm) by the grazing herd. The herd was then relocated, leaving that pasture ungrazed for the remainder of the grazing season. Grass samples adjacent to 24 fecal pats per pasture were collected every 3 weeks after fecal deposition

 The field study was conducted from June to October 2019 and the study sites were visited at 3-week intervals for sample collection, starting from the third week after fecal deposition. Figure 1 illustrates the detailed experimental design that was used for all three farms. The total sampling period was from 3 weeks until 12 weeks after fecal deposition (except for pastures 1 and 2 on farm 3). The sampling dates for each pasture are shown in Additional file 1: Table S1. The experimental design allowed us to study the population dynamics of free-living GINs on each pasture for the remainder of the grazing season without any further contamination from grazing cattle.

**Fig. 2:**
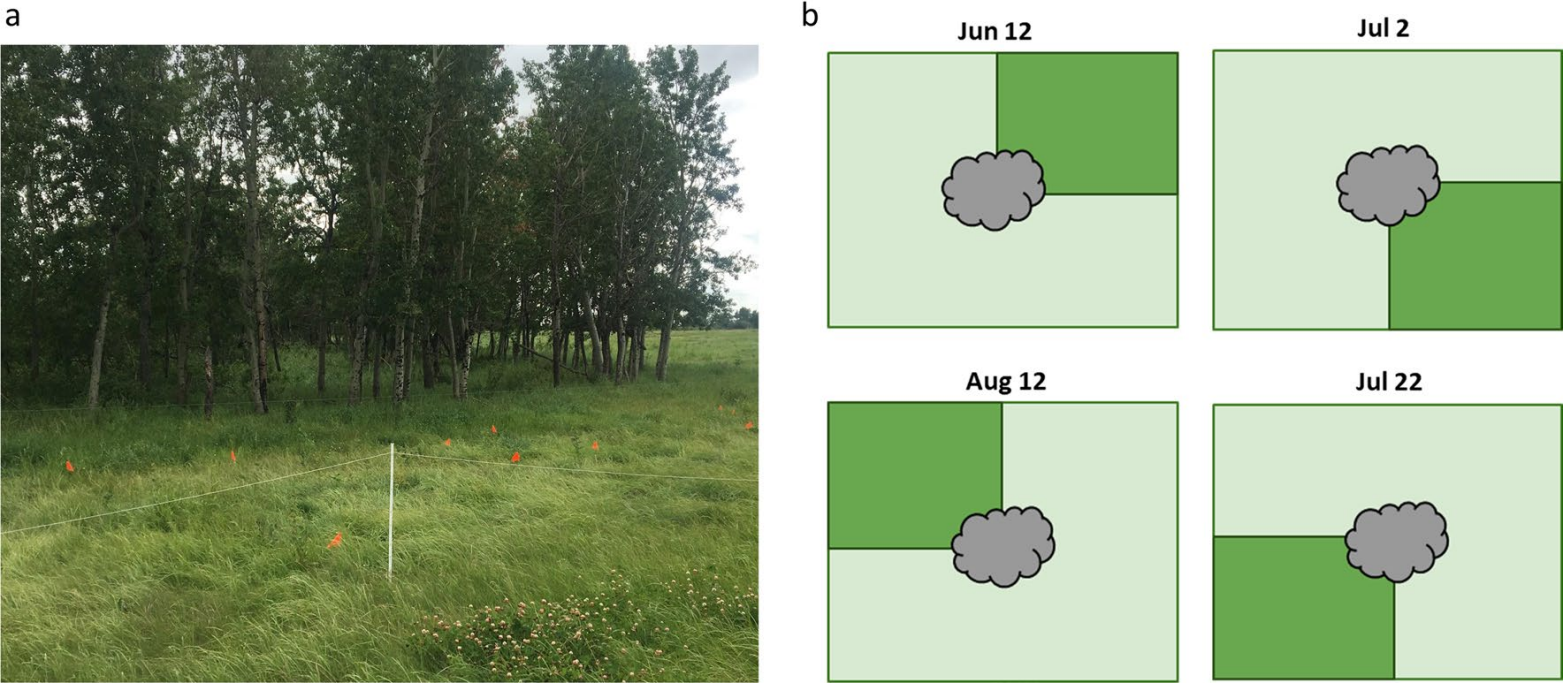
**a** On each pasture, 24 fecal pats were labelled with identification flags so that grass samples could be collected every 3 weeks from around the same pat. Fencing was set up to protect the sampling area from wildlife. **b** Schematic of the sampling method. Each sampling visit, grass samples were collected from one of the four 30 × 30-cm quadrants surrounding a pat. The quadrants were cut in clockwise order over the study period

On each pasture, grass sampling was performed around 24 random fecal pats in decent condition. Each fecal pat was labelled with an identification flag to allow the collection of grass from one of four 30 × 30-cm (900-cm^2^) quadrants immediately adjacent to the pat every 3 weeks throughout the grazing season. During each sampling visit, grass on one of the four quadrants adjacent to each pat was cut to ground level with a pair of grass shears (Fiskars, Finland) (Fig. 2a), on the basis that the vast majority of L3 migrate no further than 30 cm from deposited feces [28]. Sampling was conducted clockwise around each fecal pat. In order to keep the laboratory work to a manageable scale, on each sampling occasion, grass samples from four pats were pooled to give a total of six sampling units per pasture. Material from around the same four pats was pooled on each sampling occasion. A total of 864 grass samples (equivalent to 216 sampling units) were collected during the study period. We estimated the total number of L3 migrating from the fecal pats onto the corresponding area of grass using this quadrant sampling technique. The study area was protected by fencing to prevent disruption by wildlife (Fig. 2b). All of the grass samples were stored in sealed plastic bags during transport and analyzed within 24 h. At the end of the study, the 24 labelled pats were collected from five of the nine pastures (two pastures on farm 2 and all three pastures on farm 3), to examine how many L3 remained in the pats. Pats were not collected from all nine pastures because of labour and scheduling issues.

### Recovery of L3 from grass and fecal samples

Grass and fecal L3 recovery methods were validated by spiking 100 g of parasite-free grass with around 1000 L3 (Additional file 2: Text S1). The recovery rates of the grass and fecal L3 protocols were 22.0% and 14.4%, respectively, and were applied to the calculation of available L3. Grass samples were processed following a flotation and sieving protocol, as described by Wang et al. [13]. The total number of L3 that migrated from one fecal pat onto grass (*L3*g) was calculated as follows:

$${\text{L}}3{\text{g}} = \frac{{{\text{L}}3{\text{s }} \times { }4{ }}}{{4 \times { }0.22}}$$

where* L3*s is the number of L3 in each sampling unit.* L3*s is multiplied by 4 as a result of the quadrant-type sampling technique, where only 25% of the total grass around each fecal pat was collected on a sampling occasion; the product is then divided by the grass L3 recovery rate (0.22) multiplied by 4, as each sampling unit contained L3 from four fecal pats.

At the end of the study, each pat collected from the five pastures was processed individually and thoroughly mixed with a kitchen blender (72-oz 1500-W Ninja Pulse Control Blender; USA). A 30-g aliquot was processed to harvest L3 using a modified Baermann technique [29]. The total number of L3 in each fecal pat (*L3*f) was calculated as follows:

$${\text{L}}3{\text{f}} = \frac{{{\text{L}}3{\text{a }} \times {\text{ Wt }}}}{{{\text{Wa }} \times { }0.144}}$$

where* L3*a is the number of L3 per aliquot,* W*a is weight of the aliquot,* W*t is the weight of the whole fecal pat from which the aliquot was derived; 0.144 is the fecal L3 recovery rate.

Trichostrongylid L3 were distinguished from free-living nematodes based on key morphological features such as the presence of a sheath tail extension and filament [30]. The number of L3 in a 1-ml aliquot was determined using a counting chamber (Sedgewick Rafter, UK) under ×4 magnification, and the whole sample population on each collection date was fixed in 70% ethanol for molecular analysis.

### ITS-2 ribosomal DNA nemabiome metabarcoding

Nemabiome metabarcoding was performed as previously described [21]. In brief, a commercial kit (DNeasy PowerSoil; Qiagen, Hilden, Germany) was used to extract DNA from L3 recovered from environmental samples of feces and grass after they had been pooled by pasture and date. The ITS-2 ribosomal DNA (rDNA) target was amplified by polymerase chain reaction (PCR), and libraries prepared and deep sequenced as previously described [13]. In brief, a 350-bp fragment encompassing the rDNA ITS-2 was amplified by PCR from a 1:10 dilution of genomic DNA template. A second round limited-cycle PCR was performed to add combinatorial barcoding adapters to allow amplicons from many populations to be pooled and then sequenced on an Illumina MiSeq Sequencer using a 500-cycle pair-end reagent kit (MiSeq Reagent Kits v2, MS-103–2003; Illumina, San Diego, CA), and a mean read depth of 20,458 (range 9,068–31,196) was generated. Further details of the protocols are available at www.nemabiome.ca.

### Bioinformatic analysis

Short-read sequence data were analysed using a bioinformatic pipeline based on DADA2 [31]. Firstly, raw paired-end reads were pre-filtered and primers were removed using the cutadapt function. Then read filtering was used as a quality control process: non-structural reads were removed from the reads by the pipeline; the settings allowed two and five expected errors for forward and reverse reads, respectively. Forward and reverse reads were either merged or, if there was insufficient overlap, concatenated. An amplicon sequence variant (ASV) table was then created and chimeras removed. A recently developed database of nematode rDNA ITS-2 reference sequences was used [32]. Finally, IdTaxa was used as the classification algorithm to classify the ASVs from the pipeline at the highest resolution using the database. We used a classification confidence of 60% as the threshold when assigning the ASVs to species level, as suggested by Murali et al. [33]. Detailed information regarding this protocol can be found at https://www.nemabiome.ca/dada2_workflow.html.

### Statistical analysis

The daily mean precipitation and temperature were calculated from the meteorological data that were recorded every 15 min, following the downloading of data from the on-farm weather stations. A non-parametric Wilcoxon–Mann–Whitney test was applied to compare the daily precipitation and temperature for each of the nine sampling pastures over each specific sampling period.

The total number of L3 (which had migrated from the feces) on the grass around each pat was determined from the L3 count, as above. The total number of L3 was then converted to the number of L3 of each individual GIN species based on the relative quantitative data derived from the nemabiome analyses. To test the effect of environmental factors on the number of L3 of *C. oncophora* and *O. ostertagi* present on the grass surrounding the fecal pats, individual species’ data for all nine pastures were combined in a single dataset. L3 abundance at each collection point was log transformed and the data for each individual species analyzed in a separate multiple linear regression with the significance of independent variables determined by ANOVA. We selected the best model from the set of candidate models using a stepwise Akaike information criterion approach [34]. It was assumed that 3 weeks was the maximum development time from egg to L3 stage under the conditions observed in the study, so the independent variables were Mean temperature 3 weeks before sample collection, Total precipitation 3 weeks before sample collection, Total precipitation 1 week before sample collection, Days after egg deposition, and Pasture. Tukey’s post hoc test was applied to significant outcomes of the categorical variable (Pasture). The statistical analyses were carried out in RStudio [RStudio Team, 2016, Integrated Development for R; RStudio, Boston, MA (http://www.rstudio.com/)].

## Results

### Grass L3 count and corresponding meteorological data

**Fig. 3:**
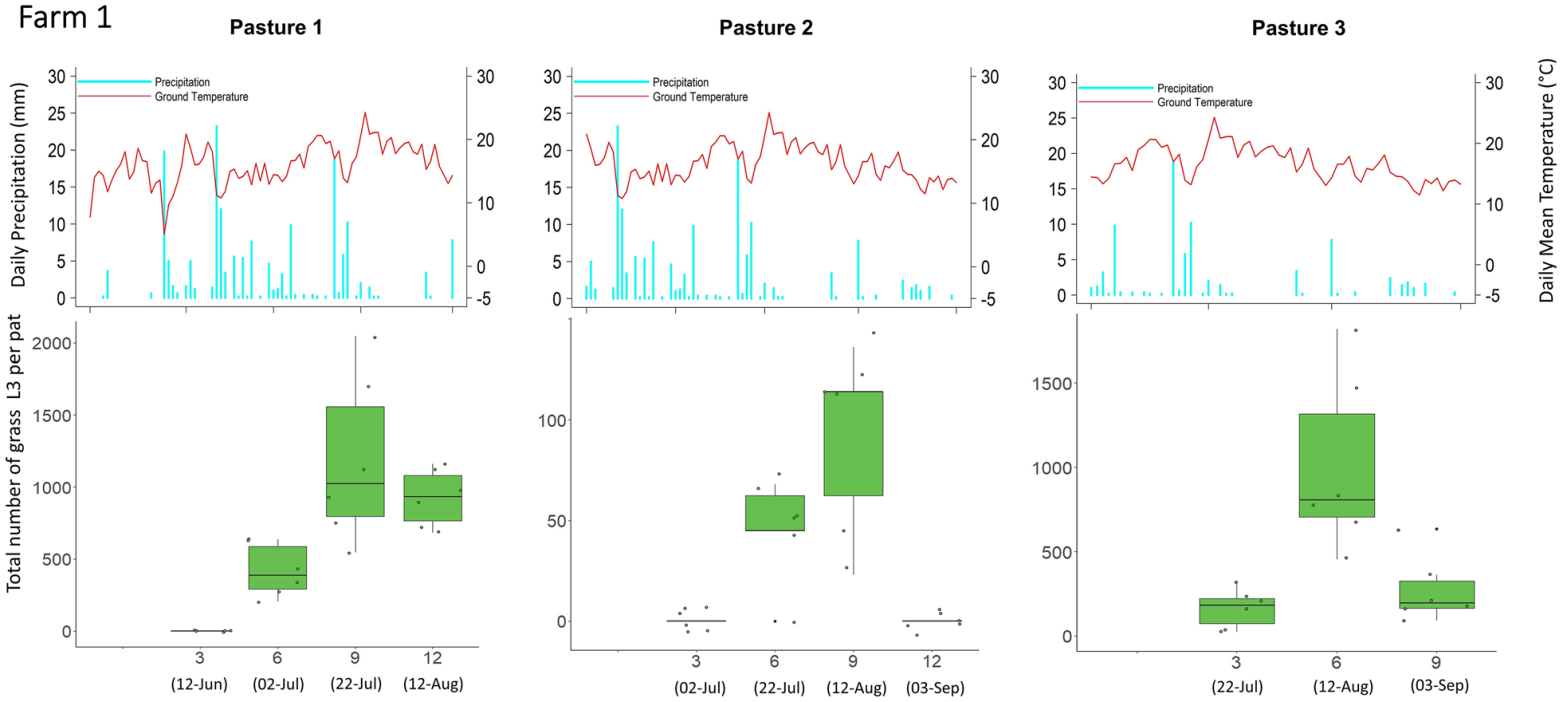
Infective third-stage larval (*L3*) count from grass samples taken adjacent to fecal pats (*lower panels*) with corresponding daily precipitation and daily mean ground temperature (*upper panels*) for farm 1. At each sampling time, the six data points correspond to six sampling units (four fecal pats per sampling unit). The *x*-axis on the lower plots indicates the number of weeks after fecal pat deposition, with sampling dates in parentheses. Each pasture was grazed only once for a 2- to 9-day period. Pasture 3 was not sampled at week 12 because the flags that identified the pats had been removed by wildlife

**Fig. 4:**
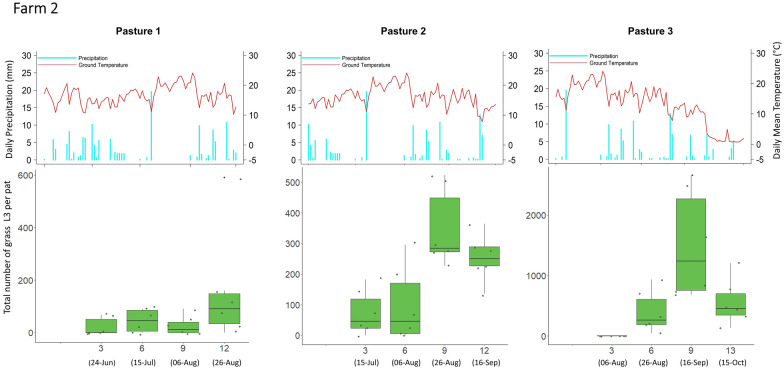
L3 count from grass samples taken adjacent to fecal pats (*lower panels*) with corresponding daily precipitation and daily mean ground temperature (*upper panels*) for farm 2. At each sampling time, the six sampling data points correspond to six sampling units (four fecal pats per sampling unit). The *x*-axis on the lower plots indicates the number of weeks after fecal pat deposition, with sampling dates in parentheses. Each pasture was grazed only once for a 2- to 9-day period

**Fig. 5:**
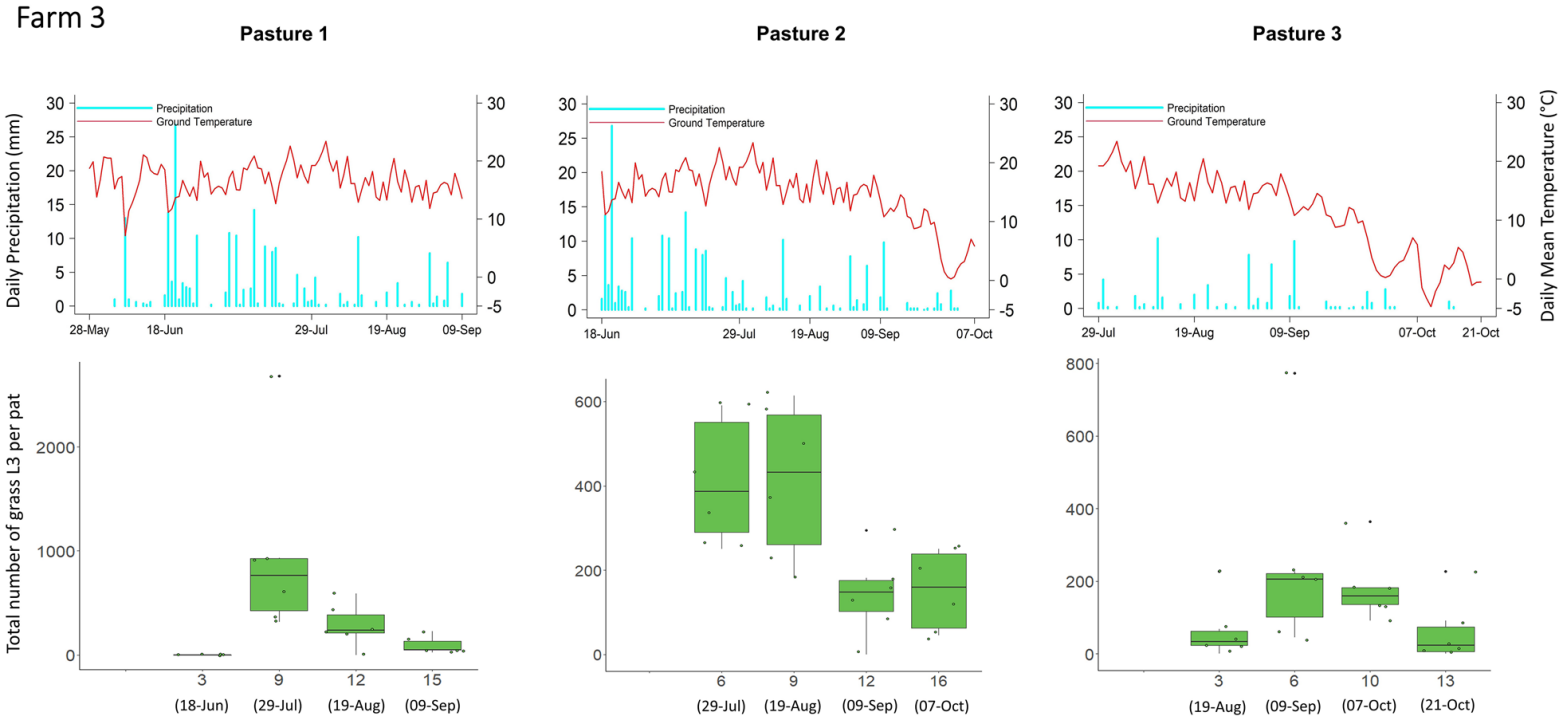
L3 count from grass samples taken adjacent to fecal pats (*lower panels*) with corresponding daily precipitation and daily mean ground temperature (*upper panels*) for farm 3. At each sampling time, the six sampling data points correspond to six sampling units (four fecal pats per sampling unit). The *x*-axis on the lower plots indicates the number of weeks after fecal pat deposition, with sampling dates in parentheses. Each pasture was grazed only once for a 2- to 9-day period. Pastures 1 and 2 were not sampled at week 6 and week 3, respectively, because of scheduling issues

Overall, and with few exceptions, the mean grass L3 count per fecal pat across all nine pastures was lowest at week 3 (mean = 36 L3/pat), followed by an increase at week 6 (mean = 311 L3/pat), then the peak at week 9 (mean 562 L3/pat), followed by a decrease at week 12 (mean = 314 L3/pat). Results for each pasture with corresponding meteorological data recorded during the study period are shown in Figs. 3, 4 and 5.

### Comparison of daily meteorological data between pastures

**Fig. 6:**
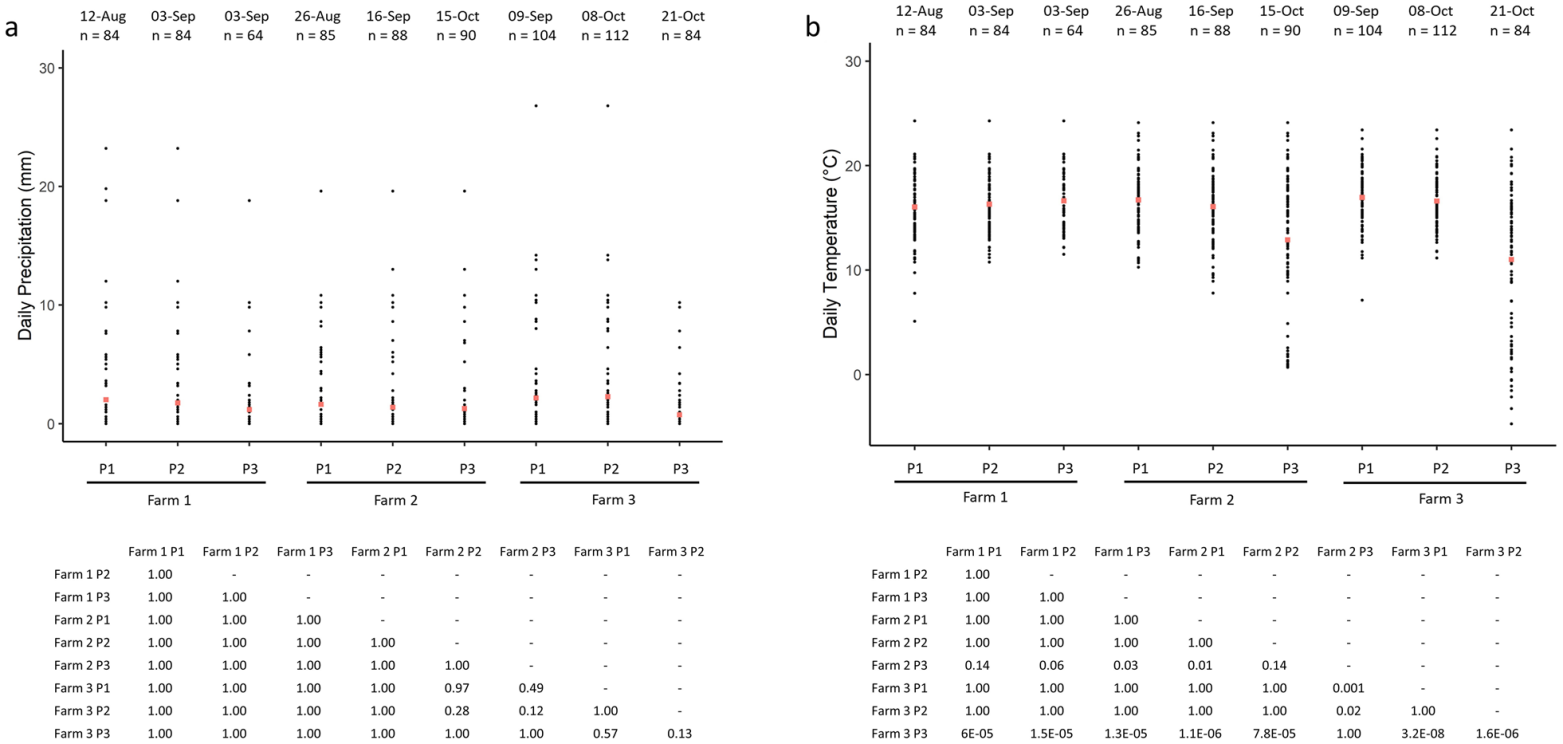
Scatter plot of daily precipitation (**a**) and temperature (**b**) for each of the nine study pastures (*P1*,* P2*,* P3*; three pastures per farm) over the entire sampling period.* Red dots* indicate the mean value for each pasture.* Dates* indicate the final sampling date for each pasture, so that the temporal changes between pastures can be reflected. Mean daily temperature and precipitation for the whole study period were 15.42 °C and 1.63 mm, respectively. The *P*-values of the pairwise comparisons are tabulated below the scatter plots. *n* Number of data points for each pasture

There was no statistically significant difference in overall daily precipitation between any of the pastures. The ground temperatures of pasture 3 on farm 2 and of pasture 3 on farm 3 were statistically significantly lower than those of the other pastures (Fig. 6).

### Fecal L3 count at the end of the study

The mean number of L3 remaining in each fecal pat, and the proportion of total L3 migrating out of the fecal pat for that pasture, are shown in Table 2. For all five pastures from which fecal pats were sampled, the overall mean number of larvae that remained in the pats and recovered from the adjacent grass at the last sampling point were 5416 (SD ± 7468) and 246 (SD ± 165), respectively .

**Table 2:** Mean (± SD) fecal infective third-stage larval (L3) count for five pastures on the last sampling date

	Fecal L3 count^a^	Grass L3 count^b^
Farm 2, pasture 2	1846 (3649)	254 (70)
Farm 2, pasture 3	20,274 (20,544)	557 (347)
Farm 3, pasture 1	361 (824)	91 (75)
Farm 3, pasture 2	1859 (4642)	144 (90)
Farm 3, pasture 3	2738 (3878)	182 (87)

Sample size for fecal and grass groups is 24 and 6, respectively

^a^Fecal L3 count is expressed as the total number of L3 per fecal pat at the time of collection

^b^Grass L3 count is the estimated total number of L3 on the grass surrounding each pat on the last sampling date

### GIN species-specific pasture L3 counts over the grazing season interpolated from ITS-2 rDNA metabarcoding data

**Fig. 7:**
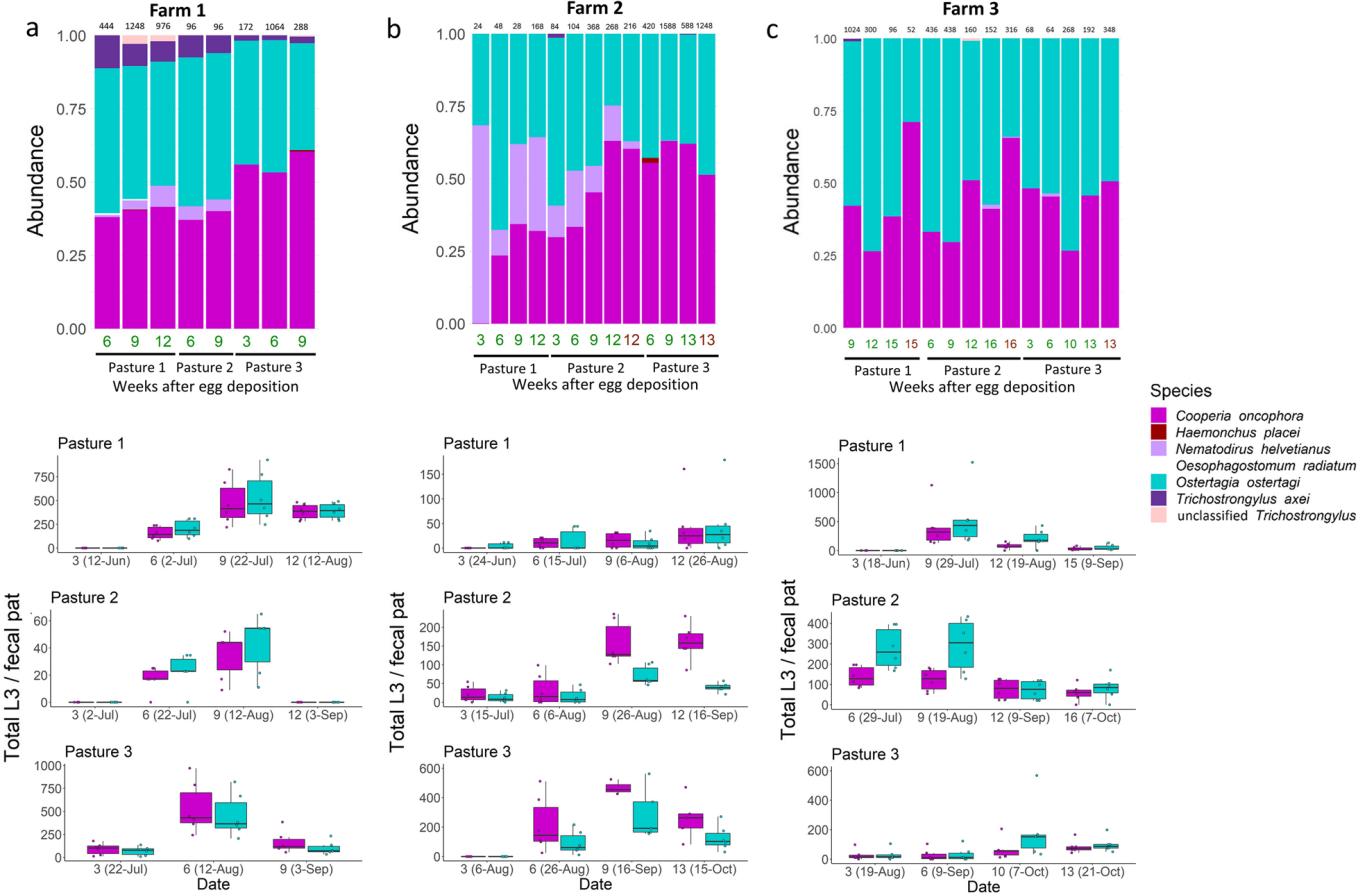
Relative species abundance in samples taken from farm 1 (**a**), farm 2 (**b**) and farm 3 (**c**) as determined by internal transcribed spacer-2 ribosomal DNA nemabiome metabarcoding of L3 populations pooled by sampling date and pasture. On the x-axis, sample numbers in* green* indicate grass samples, while those in* brown* indicate fecal samples. The number of L3 in each pool from which genomic DNA was isolated is shown above each bar. The bar charts show pasture L3 count for each species as determined by incorporating metabarcoding results with overall pasture L3 count

 The pasture L3 population of each GIN species was estimated from the total L3 values by interpolation using the GIN species proportions determined by ITS-2 rDNA nemabiome sequencing (Fig. 7). The most prevalent species on all farms throughout the grazing season was *O. ostertagi*, followed by *C. oncophora*. *Nematodirus helvetianus* and *Trichostrongylus axei* were also detected, but only at low proportions (means of 4.2% and 1.6%, respectively). Similar proportions of *O. ostertagi* and *C. oncophora* L3 were isolated for all three farms and throughout the grazing season. The overall grass count of *O. ostertagi*, *C. oncophora*, *N. helvetianus* and *T. axei* per fecal pat was 165, 169, 9 and 8, respectively.

### Relationships between climate variables and pasture L3 levels

Multiple linear regression was applied to *C. oncophora* and *O. ostertagi* datasets to test the association of five predictor variables with pasture L3 count of each species. In the case of *C. oncophora*, the predictor variables explained a significant amount of variance in the L3 count [*F*_(13, 148)_ = 19.14, *P* < 0.0001,* R*^2^ = 0.63] showing that Total precipitation 1 week before sampling and Days after egg deposition contributed significantly to pasture larval count. However, Mean temperature 3 weeks before sampling and Total precipitation 3 weeks before sampling were not statistically significant predictors (Table 3). Tukey’s post hoc honest significant difference (HSD) test was used to compare *C. oncophora* L3 harvest between pastures of the same farm. On farm 1, significantly fewer L3 were recovered from pasture 2 than from pastures 1 and 3 (*P* < 0.001). On farm 2, significantly more L3 were recovered from pasture 3 than from pastures 1 and 2 (*P* < 0.001). There was no significant difference in the L3 harvest between pastures on farm 3. The predictor variables also explained a significant amount of variance in the *O. ostertagi* L3 count [*F*_(13, 148)_ = 11.7, *P* < 0.0001,* R*^2^ = 0.51]. While Mean temperature 3 weeks before sampling, Total precipitation 1 week before sampling and Days after egg deposition contributed significantly to the model, Total precipitation 3 weeks before sampling did not (Table 3). In contrast with *C. oncophora*, Mean temperature 3 weeks before sampling was significantly associated with *O. ostertagi* L3 harvest. Tukey’s post hoc HSD test was used to compare *O. ostertagi* L3 harvest between the pastures of the same farm. On farm 1, significantly fewer L3 were recovered from pasture 2 than from pastures 1 and 3 (*P* < 0.001). On farm 2, significantly more L3 were recovered from pasture 3 than from pasture 1 (*P* < 0.001). On farm 3, significantly more L3 were recovered from pasture 2 than from pasture 3 (*P* = 0.014).

**Table 3:** Results of multiple linear regression on the number of L3 recovered from the grass surrounding fecal pats in relation to climate variables for the two main nematode species found in the study, *Cooperia oncophora* and *Ostertagia ostertagi*

	*C. oncophora*		*O. ostertagi*	
	Regression coefficient	*P*-value	Regression coefficient	*P*-value
Mean temperature 3 weeks before sampling	− 0.0215	0.540	− 0.0317	0.019*
Total precipitation 3 weeks before sampling	− 0.0117	0.897	− 0.0116	0.105
Total precipitation 1 week before sampling	0.02326	0.008***	0.01631	0.005**
Days after egg deposition^2^	− 0.00039	0.006***	− 0.00044	< 0.001***
Intercept	5.215		5.790	
*R*^2^ (*R*^2^ adjusted)	0.63 (0.6)		0.51 (0.46)	

Mean temperature in the 3 weeks prior to sampling was also included, but its effect was not significant

* *P* < 0.05, ** *P* < 0.01, *** *P* < 0.001

## Discussion

A detailed understanding of the population dynamics of GINs throughout the grazing season is crucial to support evidence-based control strategies. In this study, we investigated the pasture larval ecology of cattle GINs in the northern continental climate zone of western Canada. The number of L3 that appear on grass is the net effect of multiple aspects of the free-living phase of the parasite’s life cycle, from development inside the egg to hatching, development from L1 to L3, and migration onto pasture [28]. Our experimental design allowed us to monitor the free-living infective L3 accumulating on the grass surrounding the fecal pats over time during the summer grazing season. Since macroparasites such as GINs commonly have an aggregated distribution among a host population [35], and spatial distribution on pastures [36], substantial variability can be detected in field studies. To mitigate this, our study design was focused on the grass surrounding identified fecal pats rather than across the pastures in general, with repeated sampling of the grass surrounding a total of 24 fecal pats per pasture, for three separate pastures on each of the three farms.

 One striking result was that the grass L3 count peaked very consistently at around 9 weeks after fecal pat deposition on seven of the nine pastures sampled; small numbers of L3 appeared at 3 weeks after egg deposition on these pastures, and there was a decline in the number of L3 after 9 weeks. The remarkably similar timing of the peak of infective larvae between pastures is of particular note since both moisture and temperature have been shown to play an important role in larval development and the migration of L3 from dung onto herbage [37]. Temperature and precipitation were very similar between the pastures, and this may account for the similarity in the timing of their pasture larval peaks. Two pastures which were monitored later on in the grazing season and into October, experienced lower overall ground temperatures during the sampling period, thus, the average ground temperatures were lower after the period of larval development in the pats on these pastures.

Larval counts on grass may have declined after 9 weeks due to a combination of reduced larval activity, a reduced density of larvae remaining in the fecal pat, and the overall lower level of precipitation later on in the grazing season (Fig. 6). The L3 that had already translocated onto pasture could have either suffered high mortality from desiccation [38] or migrated downwards into the soil [39]. Multiple linear regression showed that accumulated rainfall 1 week prior to sample collection had a significant impact on the pasture L3 population, but accumulated rainfall 3 weeks prior to sample collection did not, suggesting that the pasture L3 populations depend significantly on short-term microclimatic conditions for horizontal migration onto pasture rather than longer term microclimatic variation. For GINs of sheep, rainfall is crucial for their migration out of feces [27]; earlier in the cycle, moisture within the feces is adequate for development except under the driest conditions [40], while free water is not needed for vertical migration onto grass [41, 42]. This situation is likely to be even more marked for GIN in cattle, whose larger fecal pats retain moisture for longer periods but form a thicker surface crust of dried feces, which hinders larval migration. More research is required on the climatic constraints of larval migration from cattle dung to herbage. Empirically, however, it is clear that periods of dry weather can delay larval availability in temperate (e.g. [43]) and semi-arid regions (e.g. [44]), while the sequestration of larvae in dung in the absence of rainfall could explain the lack of a 9-week peak in L3 abundance on pasture 1 of farm 2. Temperature over the previous three weeks was associated with L3 availability for *O. ostertagi* but not for *C. oncophora*, and might indicate a greater temperature sensitivity of developing larvae within the pat for the former species.

 Our results suggest that peak larval abundance occurs approximately nine weeks after fecal pat deposition in the northern continental summer, and provide supporting empirical data for basic risk assessments and grazing management advice. For example, in the rotational grazing systems of western Canada, moving cattle to new pastures within three weeks is likely to largely eliminate the risk of reinfection with GINs, while slightly longer periods would allow moderate re-infection (e.g. to preserve refugia post-treatment), while still preventing a peak of L3 density occurring at 6–nine weeks. Return intervals can also be determined by taking into consideration declining L3 abundance; although we did not track the fate of the L3 remaining in the dung pats after 12 weeks, this may have indicated that it was better to prolong the period of time until re-grazing was considered safe. Clearly, in cases where there are large temperature and precipitation differences to those observed here, such assumptions would not be valid, although a similar grazing interval to the one used here seems to control GIN effectively in temperate zones [45]. Predictive modelling studies are a potentially powerful way to build on this baseline information to provide guidance across a wider range of climatic conditions [46].

 The results of studies on parasite epidemiology that examine natural infections are difficult to interpret because of the difficulty of identifying the species composition of mixed field populations. Species-specific studies generally involve either placing fecal samples infected with a laboratory cultivated species into the field (e.g. [47]) or allowing artificially infected animals to graze and contaminate monitored pastures (e.g. [9]). Nogareda et al. [11] identified the species composition of adult worms at necropsy of tracer calves; however, carrying out this type of study might be problematic in some countries due to prevailing animal ethics, as well as associated costs and logistical challenges. In contrast, ITS-2 rDNA nemabiome metabarcoding can accurately quantify GIN species composition in a mixed field population, which makes the direct analysis of pasture contamination derived from naturally acquired infections more feasible. *O. ostertagi* and *C. oncophora* were the most abundant species in the present study; they were present on all of the pastures, although small numbers of *N. helvetianus* and *T. axei* were also detected on some pastures. The larval dynamics were essentially the same for *O. ostertagi* and *C. oncophora*, which suggests that a standard grazing management plan would be equally effective for both, at least from the perspective of larval ecology. Differences in the acquisition of immunity for these two species, however, may have an impact on production following re-exposure [48]

Another interesting finding of this study was the relatively large number of infective L3 recovered from fecal pats at the end of the grazing season. This result suggests that the fecal pat is an important refuge for L3 and could act as a reservoir for pasture contamination for an extended period of time. The estimated mean proportion of the L3 population that migrated out of fecal pats onto grass during the course of the grazing season was between 10.3% and 78.7%, depending on the pasture (Additional file 3: Table S2). This suggests that short intense periods of rainfall could still allow substantial numbers of L3 to leave dung pats even later in the grazing season. It also highlights the importance of further investigation into the impact of short-term weather variability on pasture infectivity and the potential role of predictive modelling in risk assessment and grazing management. Our findings on the importance of the fecal pat as a refuge and reservoir of infective L3 during the grazing season builds on our previous work which showed the importance of the fecal pat as a refuge enabling *O. ostertagi* and *C. oncophora* L3 survival on pastures over the winter in western Canada [13]. Under temperate northern European climate conditions, the mortality rates of *O. ostertagi* and *C. oncophora* L3 were substantially higher in feces than on herbage, and migration from feces was found to be little influenced by microclimate [49]. In northern continental climate zones, however, dry periods might inhibit larvae from migrating from feces, and cold weather might reduce their survival on herbage, hence increasing the relative importance of dung pats as a larval reservoir. Delayed migration of L3 from pats could alleviate infection pressure in the summer, but might also slow down the build-up of immunity [50] and increase autumn exposure and hence hypobiosis and the subsequent risk of type II ostertagiosis [45, 51]. Altered mixing of resistant and susceptible genotypes following anthelmintic treatment, due to larval sequestration and release, could also impact the effectiveness of refugia-based parasite control strategies [52, 53]. The implications of fecal larval sequestration for parasite control practices in cattle in northern continental zones could be far-reaching, and require further research in order to support advice given to producers. Changing climates in other parts of the world, for example drier summers in temperate zones [46], might also increase the epidemiological significance of this phenomenon for parasite control in the future.

## Conclusions

This detailed investigation of pasture L3 dynamics during the grazing season in western Canada considerably improved our understanding of the epidemiology of GINs in this region. On most pastures, L3 could not be recovered in large numbers until 6 weeks after fecal deposition, and the L3 count peaked at the 9th week after fecal deposition. A large number of larvae remained in the fecal pats at the end of grazing season, which suggests that fecal pats are important refuge for larvae under the climatic conditions reported here. Short-term rainfall seems to have an important effect on the migration of larvae from the fecal pat onto pasture. *Cooperia oncophora* and *O. ostertagi* were the two most predominant species, with comparable seasonal prevalence and abundance across the multiple locations examined. The results of this study can be used to design evidence-based control strategies, and to refine predictions of parasite epidemiology under changing climatic and management conditions.

## Supplementary Information

**Additional file 1:** **Table S1.** Grass sampling dates for each pasture during the whole study period.

**Additional file 2:** **Text S1.** Validation of grass L3 recovery and fecal L3 recovery protocols. Recovery rates were calculated by spiking parasite-free grass and fecal samples with around 1000 L3s.

**Additional file 3:** **Table S2.** The proportion of L3 migrating out of fecal pats over the course of the entire grazing period was estimated by* X*/(*X* + *Y*), where* X* is the sum of the mean number of L3 on grass per pat at each sampling point over the grazing season and* Y* is the mean number of L3 remaining in each fecal pat at the end of the grazing season.

## Data Availability

Data supporting the conclusions of this article are included in this published article and its additional files. All the raw sequences were submitted to the NCBI database (https://www.ncbi.nlm.nih.gov/sra/PRJNA783888) with accession number PRJNA783888.

## Abbreviations

GIN: Gastrointestinal nematode
ITS: Internal transcribed spacer
L3: Infective third-stage larvae
PCR: Polymerase chain reaction
rDNA: Ribosomal DNA
