# Supplementary material for: Seasonal epidemiology of gastrointestinal nematodes of cattle in the northern continental climate zone of western Canada as revealed by internal transcribed spacer-2 ribosomal DNA nemabiome barcoding
Source: Parasit Vectors. 2021 Dec 11;14:604. doi: 10.1186/s13071-021-05101-w (PMC8665551; doi:10.1186/s13071-021-05101-w)
Supplement: Supplementary file 2 — Additional file 2: Text S1. Validation of grass L3 recovery and fecal L3 recovery protocols. Recovery rates were calculated by spiking parasite-free grass and fecal samples with around 1000 L3s. [file 13071_2021_5101_MOESM2_ESM.docx]

The validation of any L3 recovery protocols (e.g. grass and fecal L3 recovery) can be achieved with the following steps:

1. Thoroughly mix the solution containing laboratory or field L3 passage
2. Take 1ml aliquot from the middle of the tube and count number of L3 in the aliquot
3. Repeat the above two steps 10 times and get the mean (M) and standard deviation
4. Evenly drop a certain volume of L3 solution on parasite-free faeces (or grass) to allow the spiked number of L3 to be around 1000
5. Apply the L3 recovery protocol on the spiked samples and count recovered L3
6. Repeat step four and five 10 times and get the mean (N) and standard deviation of L3 count
7. Recovery rate = N/M

Validation result in this study is shown below:

**Grass L3 recovery protocol (1017 spiked L3) Fecal L3 recovery protocol (1025 spiked L3)**

| Sample ID | L3 | Recovery Rate |
| --- | --- | --- |
| 1 | 280 | 0.28 |
| 2 | 260 | 0.26 |
| 3 | 240 | 0.24 |
| 4 | 165 | 0.16 |
| 5 | 248 | 0.24 |
| 6 | 209 | 0.21 |
| 7 | 264 | 0.26 |
| 8 | 204 | 0.20 |
| 9 | 182 | 0.18 |
| 10 | 149 | 0.15 |
| mean | 220 | 0.22 |
| std | 43 | 0.04 |

| Sample ID | L3 | Recovery Rate |
| --- | --- | --- |
| 1 | 110 | 0.11 |
| 2 | 140 | 0.14 |
| 3 | 146 | 0.14 |
| 4 | 150 | 0.15 |
| 5 | 148 | 0.14 |
| 6 | 100 | 0.10 |
| 7 | 200 | 0.20 |
| 8 | 150 | 0.15 |
| 9 | 180 | 0.18 |
| 10 | 148 | 0.14 |
| mean | 147 | 0.144 |
| std | 27 | 0.03 |
